# Normal Labour

**Published:** 1900-11-10

**Authors:** 


### NORMAL LABOUR.

During the many tedious discussions about the function of the midwife, much used to be made of the point that normal labour is a physiological act and one that can be properly performed without trained surgical assistance. In the report of the recent meeting of the New York State Medical Association,[1] we find additional proof of the difficulty of drawing the line between the normal and the abnormal; at any rate if it is to be maintained that normal labour does not require skilled and surgically trained assistance; for the title of one of the papers read was "Management of Normal Labour, including the Use of the Forceps," and the object of the paper was among other things, to show the advantage in such cases of giving an anæsthetic and of using the forceps. It is quite possible that many of the cases referred to would do quite well if left alone. On that question we do not enter. But this is clear, that if the teaching is sound that normal labour should be helped in such a manner as is suggested the rôle of the midwife is a thing of the past.

[1] New York Med. Rec., Oct. 20.

